# How SARS-CoV-2 and Comparable Pathogens Can Be Defeated in a Single Day: Description and Mathematical Model of the Carrier Separation Plan (CSP)

**DOI:** 10.3389/fpubh.2021.640009

**Published:** 2021-03-05

**Authors:** Robert Epstein, Connan Houser, Ruixiao Wang

**Affiliations:** ^1^American Institute for Behavioral Research and Technology, Vista, CA, United States; ^2^University of California, Los Angeles, Los Angeles, CA, United States

**Keywords:** Carrier Separation Plan, COVID-19, SARS-CoV-2, National Testing Day, secondary screening, coronavirus model

## Abstract

A simple, common-sense, three-component procedure—the Carrier Separation Plan (CSP)—can immediately halt the transmission of SARS-CoV-2 or a comparable pathogen, allow the safe reopening of an entire economy without the need for social distancing, and quickly eradicate the pathogen from the population (assuming the pathogen can be killed by the immune systems of the carriers). The three components are (a) nearly simultaneous self-testing for the pathogen by an entire population, followed rapidly by (b) nearly simultaneous self-isolation of carriers, and (c) secondary screening at entrances to facilities where people congregate. After a period of preparation lasting roughly 5–10 weeks, these steps could and probably should be taken in a single day. The power of this methodology has already been demonstrated in varying degrees with groups ranging in size from 1,000 to 11 million. Although this plan might seem daunting, its costs are minimal compared to the losses we have incurred by relying on half measures, and the US and other countries have the technological, logistical, and industrial capacities to implement this plan in a matter of weeks. With proper messaging during the weeks leading up to the testing, compliance in such a program is likely to be high given the potential benefits, and because participation is voluntary and testing is noninvasive, the legal and ethical issues associated with such a program are minimal – trivial, in fact, compared to those associated with imposing a months-long lockdown on an entire population. A SIRD/CSP model suggests that the single-day testing and separation procedure will substantially lower the number of infections, even if compliance with the procedure is modest. Modeling also suggests that when long-term secondary screening is added to the 1-day procedure, over time, the pathogen is eradicated from the population. This can occur even when compliance with secondary screening is itself relatively low.

The devastation caused by SARS-CoV-2 in 2020 has led to an unprecedented worldwide effort to combat the virus, as well as a call for rapid innovation and fresh perspectives. It has presented world leaders with a dilemma of epic proportions: How does one choose between people's health and economic health? (1). In the absence of effective treatments and with vaccines still unavailable in most of the world, the most impactful interventions at this time are almost certainly behavioral (2). In this spirit, we propose a simple, economical, common-sense method for defeating pathogens such as SARS-CoV-2 relatively quickly and without having to shut down essential societal systems.

All the methods that have so far been employed to fight the novel coronavirus—selective testing, social distancing, contact tracing, the closing of schools and businesses, the wearing of masks, and so on—can be considered half measures. They slow viral transmission, but they do not stop it, and because the virus can be spread by asymptomatic carriers (3, 4), these methods necessarily allow an unknown number of carriers to continue to infect other people. The widespread use of medically effective masks (FFP1 and FFP2) has recently been proposed as a way of slowing the spread of the virus, and much of the world is hoping that vaccines will defeat the virus, assuming, of course, that they will be effective against rapidly spreading new strains. We also acknowledge that a small number of countries that employed testing, social distancing, and other half-measures aggressively when the virus first appeared within their borders were able to suppress it almost completely. In much of the world, however, both deaths and cases continue to increase at an alarming rate (5, 6), even when social distancing and similar methods have been adopted (3). We thus pose a challenging question: When fighting a pandemic, is there an alternative to half measures?

## A Full-Measure Solution

**Figure 1:**
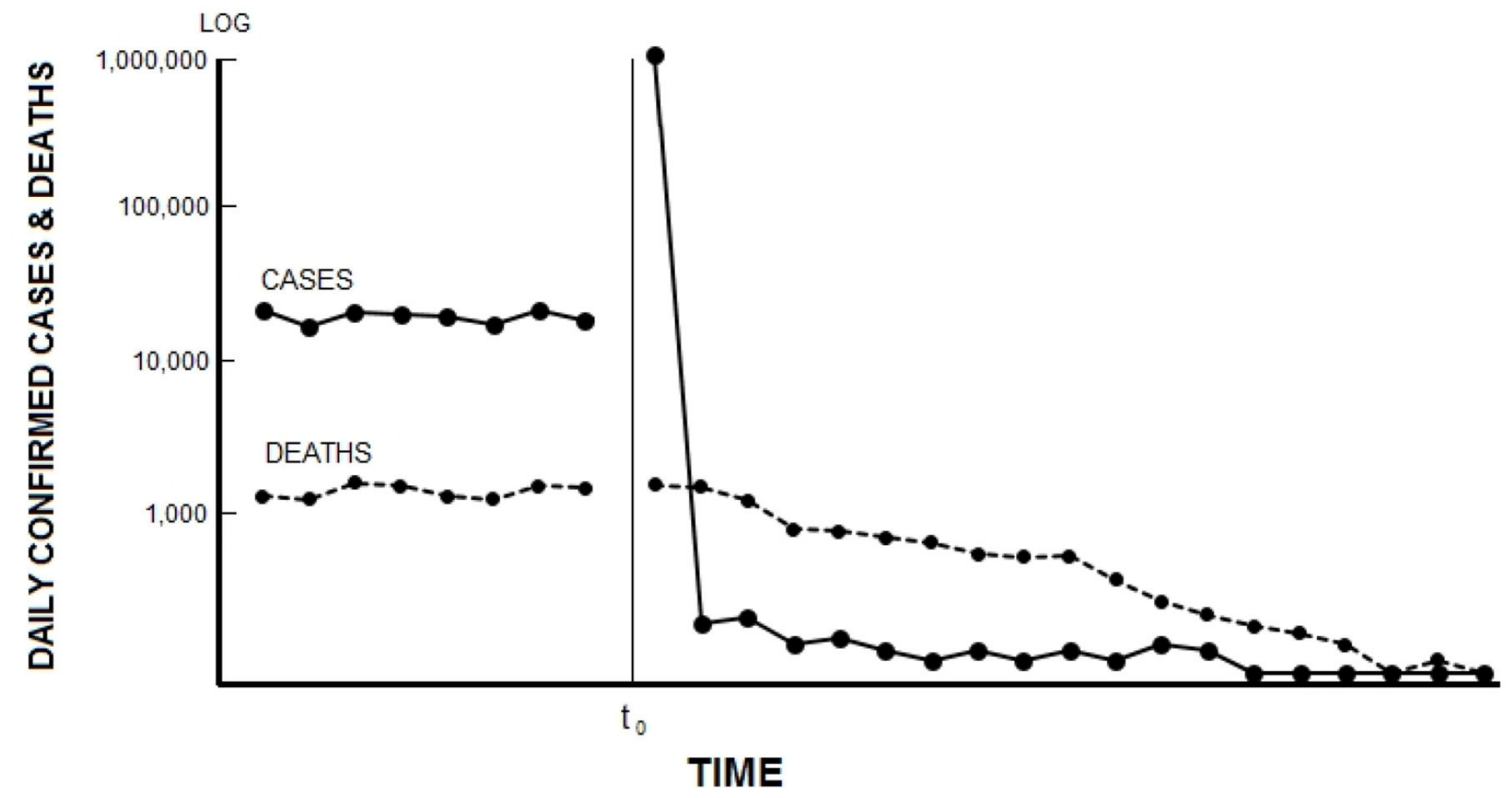
Hypothetical cases and deaths before and after National Testing Day (*t*_0_).

We submit that a full-measure solution to the novel coronavirus problem is possible and that this solution can be implemented without shuttering schools and businesses. After a period of preparation probably lasting 5–10 weeks, the solution, which we call the Carrier Separation Plan (CSP), has three main components: (1) nearly simultaneous self-testing by an entire population, followed rapidly by (2) nearly simultaneous self-isolation by those who test positive for the pathogen, and (3) secondary screening at the entrances to most or all facilities where people congregate. All three of these measures could be implemented in a single day. As a practical matter, for this plan to have its greatest possible impact—the immediate cessation of pathogenic transmission and, within a matter of weeks, the complete eradication of the pathogen from the population—all three measures must in fact be implemented in rapid succession [see Supplementary Figure 1 for further details about the procedure^1^]. The time required to eradicate the pathogen will vary according to the incubation and recovery periods of the illness caused by that pathogen. As shown in Figure 1, the number of new cases will rapidly drop to 0, whereas the number of deaths will decrease gradually over time.

The main barriers to implementing such a plan—the absence of appropriate test devices and possible noncompliance by citizens—might seem insurmountable at first, but we submit that these barriers are inconsequential when one compares them to the ongoing carnage that has resulted from half measures (7, 8). Saliva-based self-tests that detect the live novel coronavirus have already been approved or are under fast-track review by the US Food and Drug Administration (9–11). Rapid, high-volume production is also possible: A single test-device manufacturer in China is capable of producing as many as 5 billion such devices per month, with the per-unit cost dropping to between 1 and 3 cents for large production runs (12).

## National Testing Day

Regarding Compliance: Imagine that late in July, 2020, President Trump had announced that September 16th—the 400th anniversary of the day the Mayflower left England on its historic voyage to America—would be National Testing Day (NTD) in the US, and that during the weeks leading up to that day, the US Postal Service (which reaches every residential and business address in the US) would be delivering a dozen single-use self-test devices to every household in the country, as well as thousands of such devices to every school, church, and business. Over the following weeks, Trump continues to trumpet the “best-ever” advantages of the plan, and his messaging is repeated daily by every governor and mayor in the country, as well as by hundreds of thought leaders and celebrities. Among other things, Trump stresses that the testing will be *self*-testing, which means that people's privacy and autonomy are protected – important issues for Americans.

On the morning of NTD, on national television and live-streaming on the internet, Trump, his family and staff, other government leaders, and a bevy of celebrities, take a simple saliva test using a color-change device that resembles an electronic thermometer, and learn in seconds or minutes whether they are carriers of the live virus. Carriers are then urged to self-isolate immediately, and telephone operators are standing by to offer housing to carriers who lack an appropriate refuge. Trump et al. assure people that they will need to self-isolate for only the 2 or 3 weeks it will take for them to test negative (longer in some cases), and to increase compliance a cash incentive is offered to carriers willing to reveal their identities. Medical care is provided for those who need it, and even America's homeless participate, thanks to outreach efforts by social service agencies and volunteers. Actual compliance is estimated with robocalling, mass texting, mass emailing, and other survey methods.

With carriers now separated from non-carriers, on the afternoon of NTD, millions of non-carriers—perhaps more than 300 million people in the US, some of whom will have been sheltering-in-place for upwards of 6 months—are now free to get on with their lives without the need for social distancing. This method allows for the safe reopening of the entire economy in a single day, whereas current efforts at reopening economies are only partial and, in some cases, are causing a resurgence of COVID-19 (13).

The two obvious weaknesses in the plan—some people won't comply, and the test device will inevitably produce false negatives (false positives inconvenience people, but they don't adversely affect the plan)—are mitigated by the secondary screening: barrels full of test devices at the entrances to businesses, churches, and schools. If you want to be admitted, you must first test negative; if you test positive, you are sent home. By the evening of NTD, thousands of people will already have been tested a second or third time. A SIRD-type model we have developed (see below) makes specific predictions about outcomes in which both compliance and the test device are nonoptimal.

## The Feasibility of the Plan

In early November, 2020, a variant of CSP was rapidly applied to most of the population of Slovakia, which reduced new cases there by 60% in less than a week; a negative test allowed people to return to work (14). Prior to that, variants of CSP had been successfully employed with groups ranging in size from 1,000 to 11 million in Italy, South Korea, China, and elsewhere. None of these interventions was optimal from our point of view, but each variant demonstrated how rapidly and, in some cases, how completely, viral transmission can be stopped when carriers are separated from non-carriers (15–20). It's common sense, after all. In October 2020, when 12 cases of Covid-19 were detected in Qingdao, China, all 9 million residents of the city were tested over the next 5 days, carriers were separated, and transmission stopped (21). China had previously used this method to halt transmission in Wuhan, a city of 11 million residents (16). As for the feasibility of testing at entrances, Disneyland and other amusement parks are now considering doing just that (22).

To be clear: The Carrier Separation Plan, which might sound daunting at first, is actually quite feasible. It involves the rapid mass production of a simple test device, widespread distribution of the device, and lots of messaging. The plan requires us to think big and to think outside the constraints of the typical medical model, but once we leap those mental hurdles, the feasibility of the plan becomes clear. US Surgeon General C. Everett Koop leapt similar hurdles in his attempt in 1988 to get the AIDS epidemic under control: He distributed a sexually-explicit 8-page booklet to all 117 million households in the US explaining how we could immediately stop transmission of the AIDS virus simply by changing our sexual behavior. He was thinking big and thinking behaviorally, and history has proved him right; 32 years have passed since he mailed that booklet, and we still don't have an HIV vaccine.

Why 1 day? Because testing over a period of months, as we are doing now, has much in common with the Red Queen's race. It guarantees that carriers will unknowingly continue to interact with non-carriers, thus assuring continuing transmission. How can such a course of action—one that might, over time, produce hundreds of thousands of new cases and thousands of deaths—be justified when we have the means to quickly halt the spread of the virus?

We acknowledge that not every country has the resources to implement CSP. Most industrialized countries do, however, and resource-rich countries could, both in principle and as a matter of principle, assist other countries with problems of logistics, production, and messaging. Regarding the problem of the rapidity with which a pathogen can cross borders (as new strains of COVID-19 are doing now), we suggest that the same rapid test devices used in nationwide testing can also be used to keep non-porous borders safe. Where contiguous countries have porous borders, CSP will be maximally effective only if such countries are able to test their populations simultaneously.

We also acknowledge other limitations of CSP. Secondary screening will likely fail, for example, when it comes to private gatherings; a restaurant owner might have a strong financial incentive to make sure a pathogen is not spread by his or her business, but people partying in their homes have no such incentive. Moreover, testing may fail to detect potential carriers when the virus has a long incubation period. One might also conceivably object to CSP on legal or ethical grounds, but whatever those specific grounds might be, we suggest that closing schools and businesses nationwide and locking down an entire population for months—with no clear end in sight, no less—makes any concerns one might have about a single day of self-testing and a few weeks of self-isolation look relatively harmless.

The plan we are proposing does indeed require the management of an entire population for a portion of 1 day, but that is far less challenging than trying to manage an entire population for months. In the US, efforts to do so have failed, with tragic consequences for both health and the economy (23). Bear in mind that beginning the day after National Testing Day, authorities will mainly need to manage only a few miscreant individuals or groups—far easier than trying to manage an entire population—and with the safe reopening of the entire economy as the prize, those miscreants will also be under enormous pressure from friends, colleagues, and relatives to cooperate.

Given that saliva tests are non-invasive and that participation in this program is voluntary (at least as it would need to be implemented in an individualistic country such as the US), could any legitimate objections be raised? The only objection we have heard thus far is the from-the-hip comment, “That's impossible!” But this program is not only possible, it is far less complicated, risky, and costly than the destructive and demoralizing half-measure alternatives we are relying on currently. With respect to both cost and complexity, CSP is in some respects less demanding than conducting a national census, which in some countries requires face-to-face contact with every member of the population. A census, meanwhile, has no immediate benefits, but the benefits of CSP are immediate and immense.

Could the rampant partisanship that has largely immobilized the federal government in Washington, DC, prevent such a plan from being implemented? If Trump had taken the lead on this plan in July 2020, with so much at stake economically and the COVID-19-related death toll steadily climbing toward the 200,000 mark in the US at that time (now rapidly approaching 500,000), the plan would likely have had strong bipartisan support; it's a plan that truly unites the country in a common cause, after all. Regarding the compliance issue, in the US the segment of the population that would normally resist participating in a large-scale government-directed program—the ardent anti-government faction—was the core of Trump's base of supporters. To them, Trump could do no wrong, so it's possible that they would have self-tested and self-isolated with enthusiasm. Different countries will face different challenges in implementing a population-wide testing plan, but that doesn't mean such challenges can't be met.

## The Potential of this Methodology

This methodology, properly implemented, has the potential to halt transmission of a new pathogen while allowing essential societal systems to remain open (or, if they have been shut down, to reopen fully). When a pathogen can be defeated by the immune systems of the carriers, this methodology can also eradicate the pathogen from a population. When treatment is ineffective or unavailable and the pathogen kills its host, the pathogen itself is still destroyed. Even with immunocompromising pathogens such as HIV, population-wide self-testing and self-isolation can still halt transmission and allow non-carriers to continue to lead normal lives without fear. This methodology also reduces the likelihood that hospital systems will be overwhelmed, both because it stops the spread of the disease and because it helps to quantify the prevalence of the pathogen. Finally, this methodology makes the need for a vaccine less urgent, giving pharmaceutical companies additional time in which to develop a safe and effective preventative.

## Modifying a Basic SIRD Model

We examined the likely dynamics of CSP with a modified SIRD model using ordinary differential equations. Our model is divided into three parts, each of which predicts the total number of people infected per day in the US on the days immediately following NTD. We will use September 15, 2020, as the hypothetical day when NTD was implemented in the US. Actual data and projections from September, 2020, through January, 2021, were used to constrain model parameters.

Part 1 of the model shows what happens when increasingly larger proportions of the population fail to comply with plan directives and when the test device produces levels of false negatives > 0. Part 2 shows what happens when long-term secondary screening immediately follows NTD. Part 3 examines what happens when secondary screening—now renamed “institutional screening”—is employed without a National Testing Day – in other words, without first attempting to separate carriers from non-carriers nationwide.

### Part 1: National Testing Day

Part 1 of the model suggests that CSP substantially lowers the daily number of new cases even when compliance with the plan is low and even when the test device has relatively low sensitivity. This is because large-scale testing and separation quickly remove a large number of carriers from the population.

The basic SIRD model relies on four parameters – *S*, (the number of susceptible individuals in the population), *I* (number of infected), *R* (number of recovered), and *D* (number of deaths) (24, 25). We can express these variables as proportions of the population at time *t*, where *N* is the total number of people in the population:

$$\begin{array}{l} s\left(t\right)=S\left(t\right)/N\\ i\left(t\right)=I\left(t\right)/N\\ r\left(t\right)=R\left(t\right)/N\\ d\left(t\right)=D\left(t\right)/N \end{array}$$

Changes over time in these variables can be expressed as a system of ordinary differential equations:

$$\begin{array}{l} \frac{ds}{dt}=-b\;*\;s\left(t\right)\;*\;i\left(t\right)\\ \frac{di}{dt}=\;b\;*\;s\left(t\right)*\;i\left(t\right)-k\;*\;i\left(t\right)\\ \frac{dr}{dt}=k\;*\;i\left(t\right)-m\;*\;i\left(t\right)\\ \frac{dd}{dt}=m\;*\;i\left(t\right) \end{array}$$

where *b* is the disease transmission rate, *k* is the recovery rate, and *m* is the death rate.

CSP can be integrated into SIRD by modifying *b*, because *b* influences the rate at which the infected and susceptible populations grow and shrink. We accomplished this integration by multiplying *b* by the failure rate of NTD, which is:

$$\begin{array}{l} 1-{p_{st}}^{*}{p_{si}}^{*}\left(1-p_{fn}\right) \end{array}$$

where *p*_*st*_ is the proportion of the population that complies with self-testing, *p*_*si*_ is the proportion of carriers that complies with self-isolation, and *p*_*fn*_ is the proportion of false negatives produced by the test device (so its complement is device sensitivity).

We then modified the starting parameters of the model to fit Institute for Health Metrics and Evaluation (IHME) records and projections of the daily number of Covid-19 new infections in the US beginning September 15, 2020^2^. As of the date we did the curve fitting (January 31, 2021), the IHME reported that the number of new infections on September 15, 2020 was approximately 100,000, and it predicted a peak in the number of new infections of ~340,000 on December 23, 2020. Our full mathematical model, fitted to IHME data, can be executed using a downloadable R script^3^.

**Figure 2:**
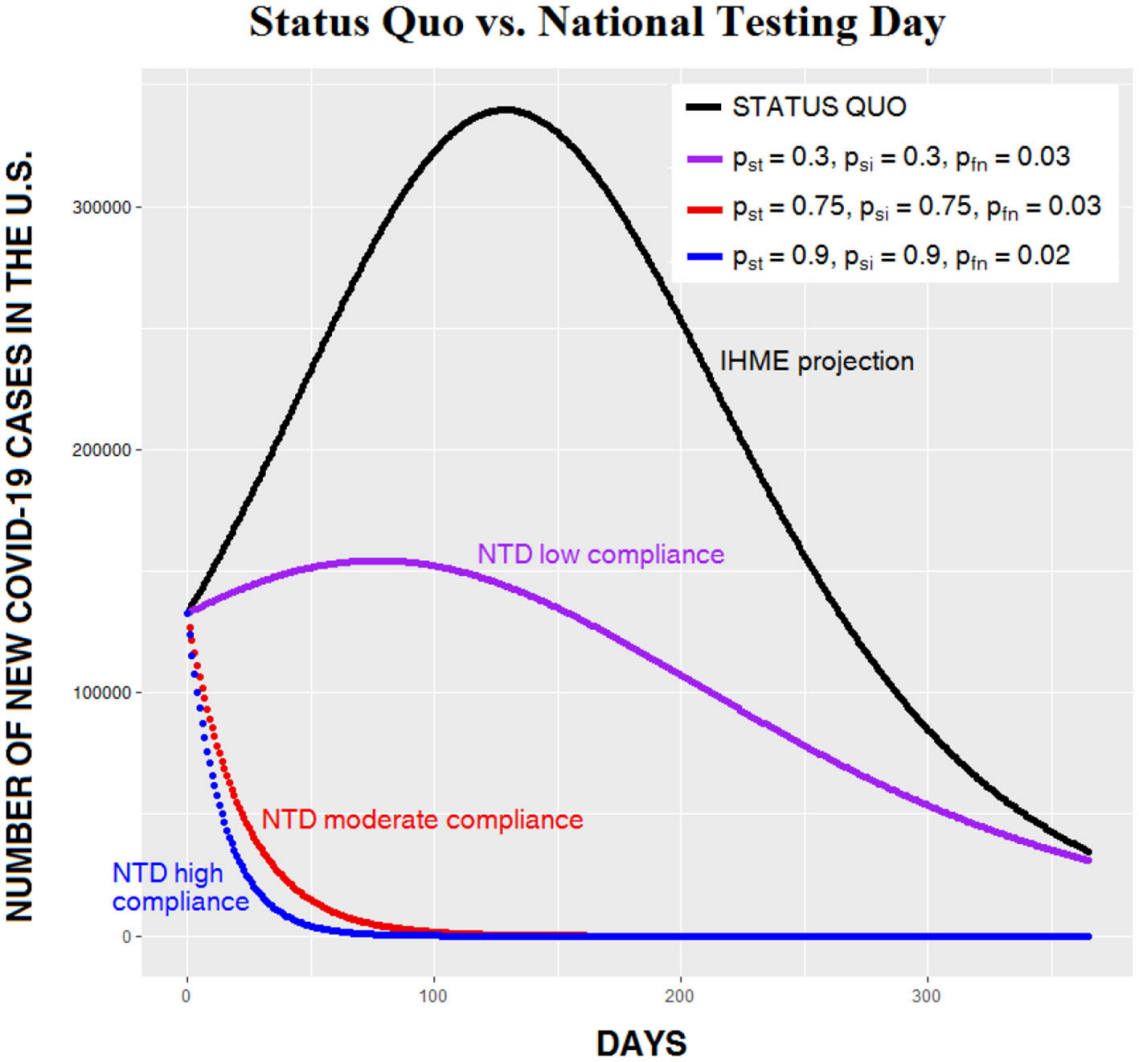
Daily number of Covid-19 cases in the US beginning September 15, 2020, given the status quo (black curve, derived from IHME data) vs. three levels of CSP effectiveness on National Testing Day (NTD). Note that when CSP success is moderate or high (red and blue curves), the increase in the number of cases evident in the other two curves does not occur.

Figure 2 compares an approximation of the IHME data (black curve) to curves showing three different levels of CSP compliance on NTD. The purple curve shows low compliance (30% compliance with self-testing, 30% compliance with self-isolation, and a test-device false negative rate of 3%). The red curve shows moderate compliance (75% compliance with self-testing, 75% compliance with self-isolation, and a test-device false negative rate of 3%). And the blue curve shows high compliance (90% compliance with self-testing, 90% compliance with self-isolation, and a test-device false negative rate of 2%).

**Figure 3:**
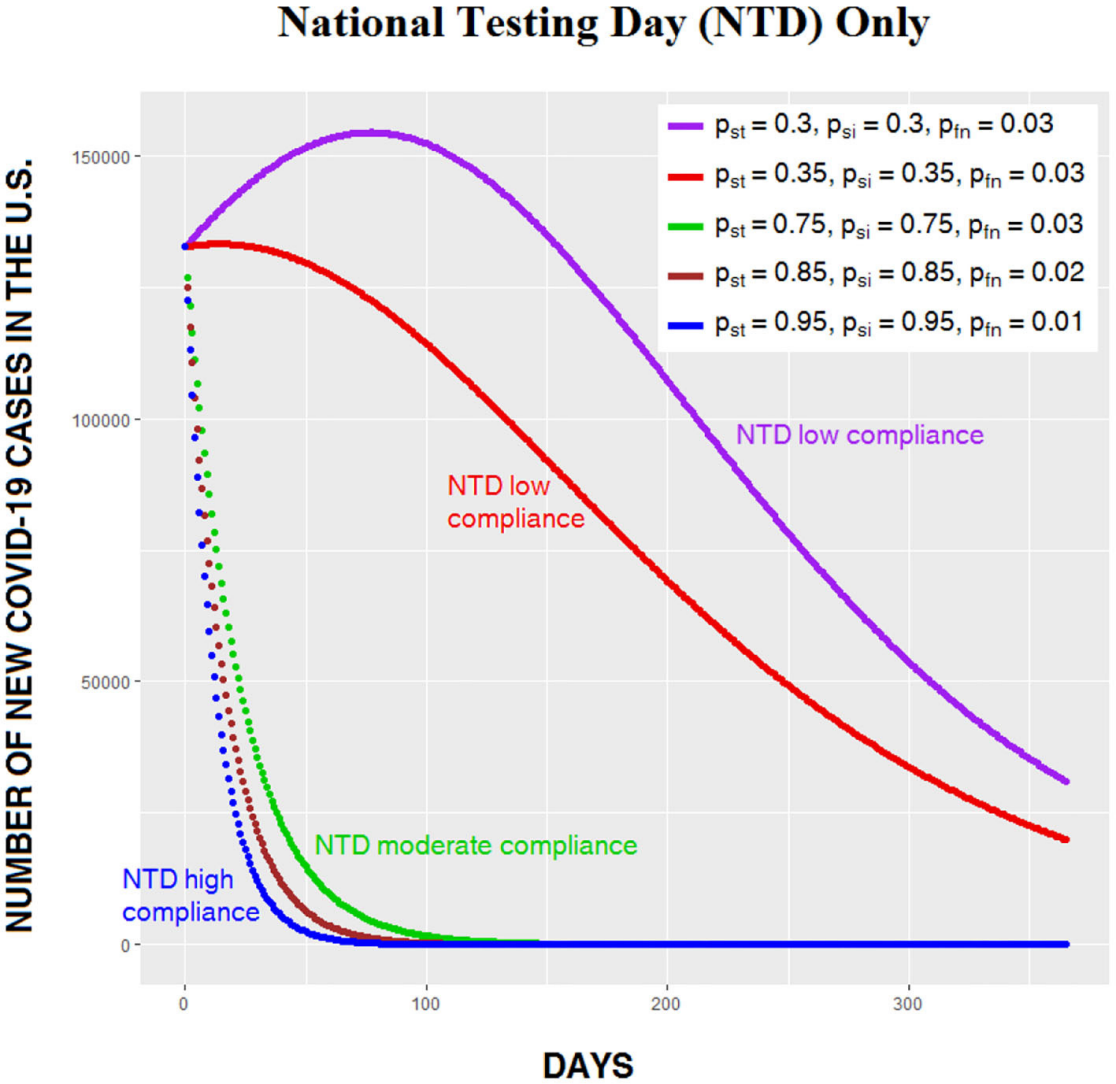
Daily number of Covid-19 cases in the US for five levels of CSP effectiveness on National Testing Day (NTD). Note the change in scale from Figure 2: The highest peak in Figure 2 is over 340,000, whereas the highest peak in Figure 3 is under 160,000.

All three levels of compliance show dramatic reductions in the number of daily infections, and both the 75 and 90% compliance levels avoid the increase in cases evident in the other curves. This is because when compliance is high, so many carriers have been removed from the general population that the recovery rate now greatly exceeds the transmission rate. As noted earlier, in the ideal case, with 100% compliance on NTD and a device with 100% sensitivity, transmission stops completely. Figure 3 compares five nonoptimal levels of CSP effectiveness on NTD. Note that in the lower three curves (describing, respectively, compliance levels of 75, 85, and 95%), the number of people infected begins to decrease immediately following NTD; the usual increase in infections is absent.

### Part 2: National Testing Day With Secondary Screening

Part 2 of the model suggests that secondary screening has the potential, over time, to eradicate the virus from the population, even when compliance with NTD directives is relatively low. Here we further modify *b* by multiplying it times *E* (0 ≤ *E* ≤ 1), the effectiveness of secondary screening, which we define as the product of *p*_*ss*_, the proportion of the population that is tested each day at the entrances to schools, churches, and businesses, and (1−*p*_*fn*_), the sensitivity of the testing device. Thus, *b* is now multiplied by a factor for NTD and by a factor for secondary screening, as follows:

$$\begin{array}{l} \left(1\;-\;p_{st}\;*\;p_{si}\;*\;\left(1\;-\;p_{fn}\right)\right)\;*\;\left(1-E\right) \end{array}$$

**Figure 4:**
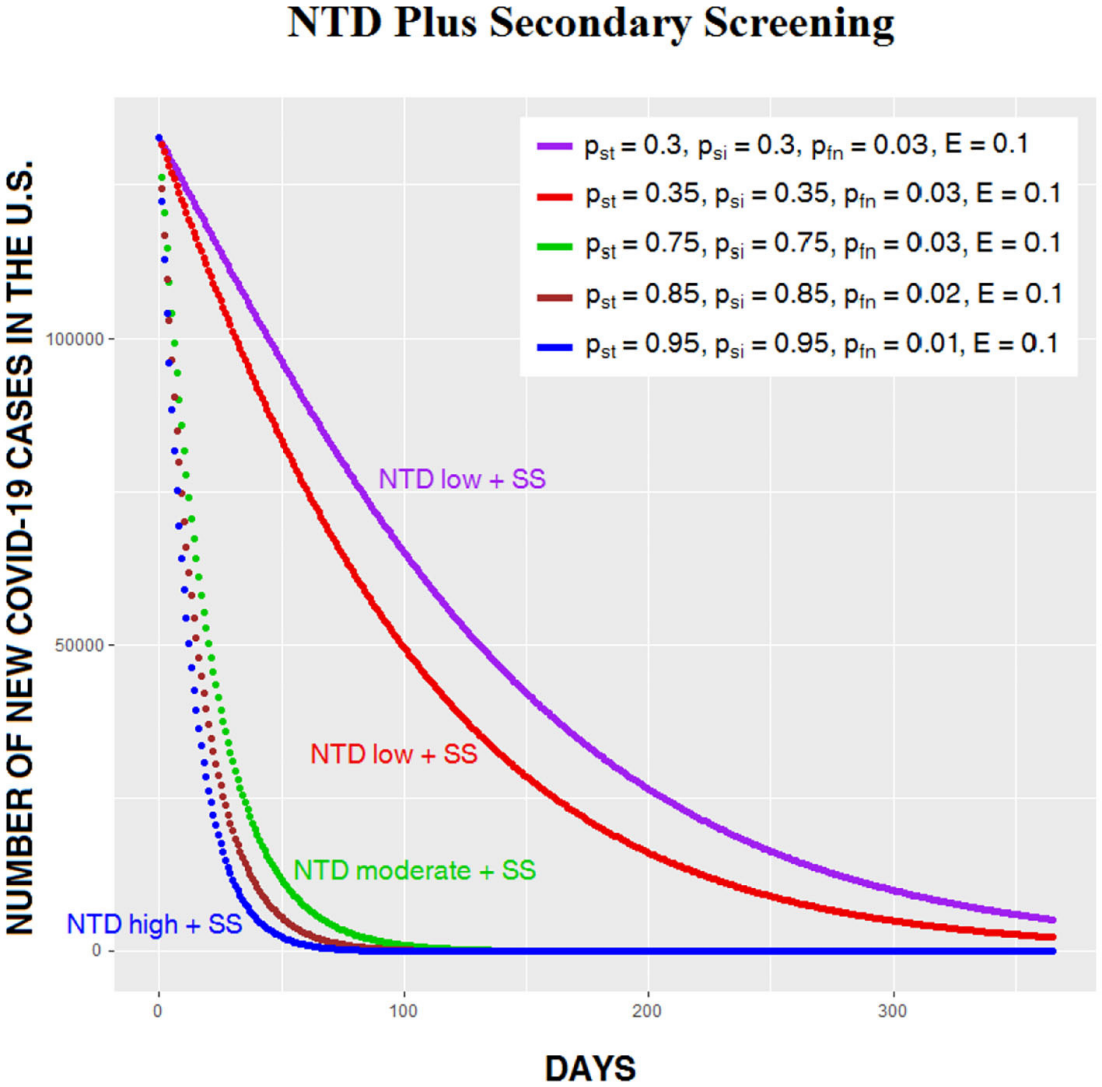
Daily number of Covid-19 cases in the US for five levels of CSP effectiveness on NTD, with secondary screening (SS) following NTD.

Figure 4 shows the same compliance levels as Figure 3, but this time with secondary screening, which flattens the curves. Even when compliance on both NTD and secondary screening are low, the number of infections begins to decrease immediately after NTD.

### Part 3: Institutional Screening Only

The apparent power of long-term secondary screening suggested in the above model raises an interesting question: Could a pathogen such as SARS-CoV-2 be defeated simply by distributing millions of test devices to places where people congregate and then encouraging the proprietors to test people before they enter and to send carriers home? In other words, could we scrap NTD and still eradicate a dangerous pathogen with a simple, large-scale institutional screening program?

**Figure 5:**
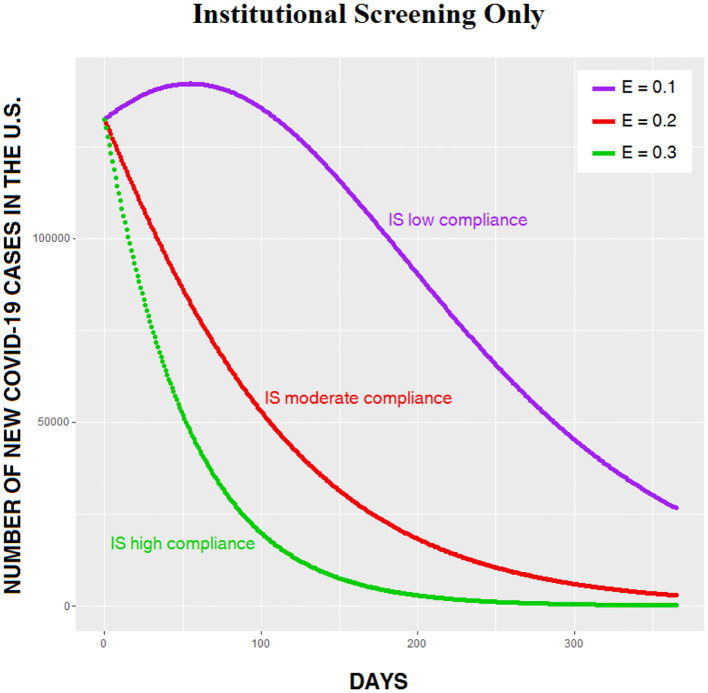
Institutional screening (IS) only, with three values of *E*.

We modeled this option by omitting the NTD factor in the calculations. Figure 5 shows three outcomes with *E* varying from 0.1 to 0.3. Needless to say, a value of 0.3, signifying that roughly a third of the population is tested every day at entrances to churches, schools, and businesses, is highly unrealistic. What's interesting here is that even low compliance (*E* = 0.1) with institutional screening eventually reduces the number of infections, although the process is slow (Figure 5, purple curve).

**Figure 6:**
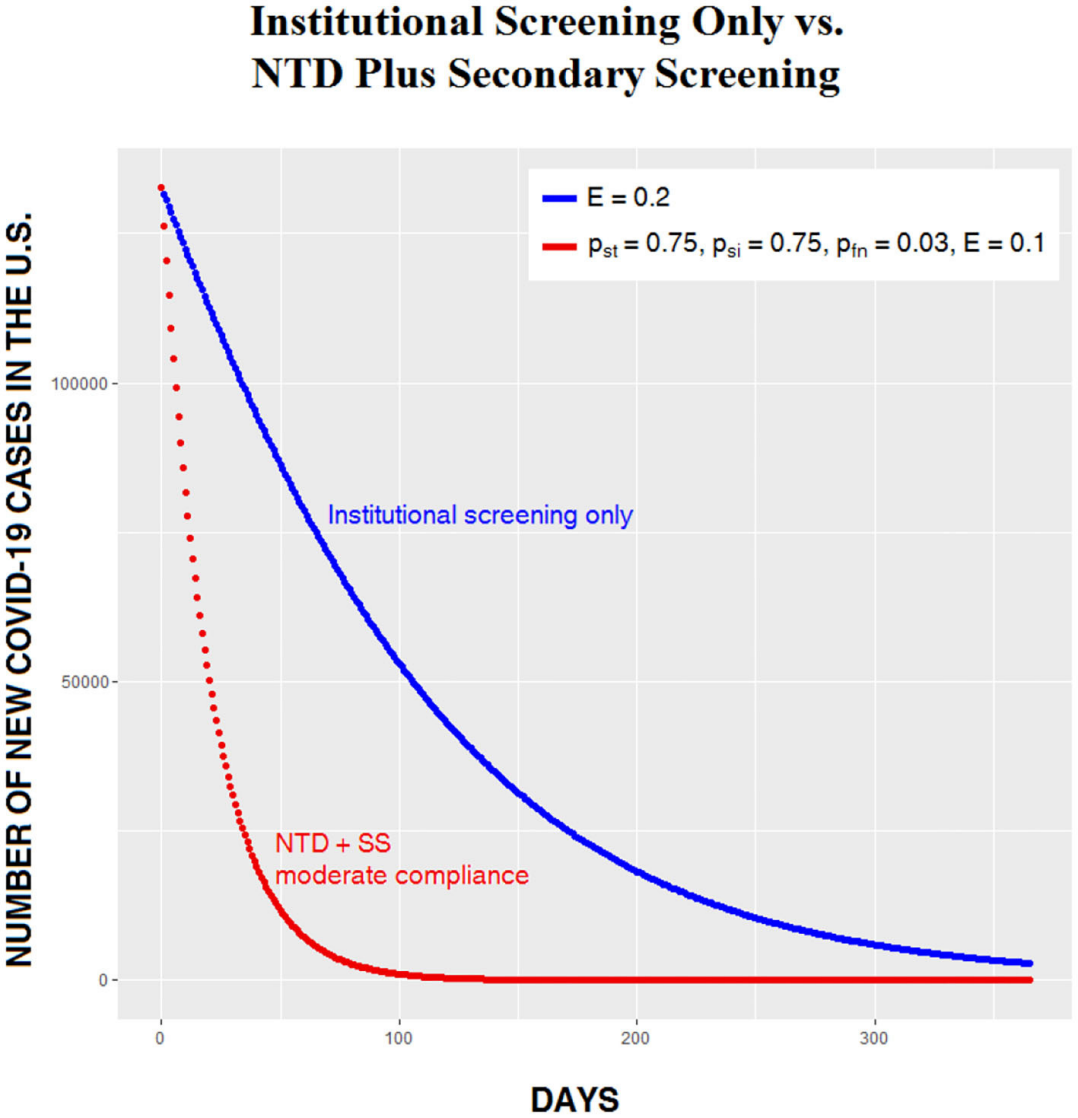
Institutional screening only, vs. NTD plus secondary screening (SS).

Given the simplicity of institutional screening, it might seem appealing as an intervention, but our analysis suggests that NTD plus secondary screening is superior to institutional screening in three ways: as a way of quickly and safely reopening the entire economy, as a means of saving lives, and as a means of eradicating the pathogen. In Figure 6, the number of daily cases generated by institutional screening given the unlikely scenario in which roughly a fifth of the population is tested daily is compared to moderate CSP compliance on NTD with a relatively inaccurate test device and low secondary screening compliance. The difference between the two curves is dramatic, with far more damage being done by an aggressive and probably unrealistic institutional screening program than by an easily achievable NTD scenario. With higher compliance on NTD, the difference between the two alternatives would be even greater. The relatively poor outcome of institutional screening alone reminds us, once again, that spreading out testing over time is a poor strategy for beating the virus, because it guarantees that large numbers of carriers—many of whom are asymptomatic—will continue to interact with non-carriers.

We conclude that the full three-step Carrier Separation Plan is more beneficial than institutional screening alone. It reduces sickness and death to a minimum, and it allows for the full and immediate reopening of society with little or no need for masks or social distancing.

## Data Availability Statement

The original contributions presented in the study are included in the article/supplementary material, further inquiries can be directed to the corresponding author/s.

## Author's Note

RE, CH, and RW propose a relatively simple and inexpensive way of stopping transmission of the virus, safely and rapidly reopening the entire economy, and, in a matter of weeks, eradicating the virus from the population.

## Author Contributions

RE has been a research psychologist for 40 years, and he has been informally proposing variants of the Carrier Separation Plan in mainstream media outlets since March, 2020 (26–33). RW is a student at UCLA, and both CH and RW are research interns at AIBRT. RE developed the plan and wrote the paper, and CH and RW developed the mathematical model. All authors contributed to the article and approved the submitted version.

## Conflict of Interest

The authors declare that the research was conducted in the absence of any commercial or financial relationships that could be construed as a potential conflict of interest.

## Footnotes

^1^https://aibrt.org/downloads/FLOWCHART-C19.1.pdf

^2^https://aibrt.org/downloads/IHME_COVID-19_projections_as_of_1-28-2021-new_cases_per_day_9-15-2020_to_5-1-2021-covid19.healthdata.orgSLASHunited-states-of-americaQviewEQinfections-testing&tabEQtrend&testEQinfections.jpg

^3^https://aibrt.org/downloads/EPSTEIN_et_al-SIRD-CSP_model-new_cases-31_Jan_2021_rev.R
